# Epigenome-Wide Comparative Study Reveals Key Differences Between Mixed Connective Tissue Disease and Related Systemic Autoimmune Diseases

**DOI:** 10.3389/fimmu.2019.01880

**Published:** 2019-08-07

**Authors:** Elena Carnero-Montoro, Guillermo Barturen, Elena Povedano, Martin Kerick, Manuel Martinez-Bueno, PRECISESADS Clinical Consortium, Esteban Ballestar, Javier Martin, María Teruel, Marta E. Alarcón-Riquelme

**Affiliations:** ^1^GENYO, Center for Genomics and Oncological Research: Pfizer, University of Granada, Andalusian Regional Government, Granada, Spain; ^2^CSIC-IBPLN, Consejo Superior de Investigaciones Científicas, Instituto de Parasitología y Biomedicina López-Neyra, Granada, Spain; ^3^IDIBELL, Bellvitge Biomedical Research Institute L'Hospitalet de Llobregat, Barcelona, Spain; ^4^Institute for Environmental Medicine, Karolinska Institutet, Solna, Sweden

**Keywords:** mixed connective tissue disease, systemic autoimmunity, genome-wide DNA methylation, epigenetics, interferon, meQTL, biomarker

## Abstract

Mixed Connective Tissue Disease (MCTD) is a rare complex systemic autoimmune disease (SAD) characterized by the presence of increased levels of anti-U1 ribonucleoprotein autoantibodies and signs and symptoms that resemble other SADs such as systemic sclerosis (SSc), rheumatoid arthritis (RA), and systemic lupus erythematosus (SLE). Due to its low prevalence, this disease has been very poorly studied at the molecular level. We performed for the first time an epigenome-wide association study interrogating DNA methylation data obtained with the Infinium MethylationEPIC array from whole blood samples in 31 patients diagnosed with MCTD and 255 healthy subjects. We observed a pervasive hypomethylation involving 170 genes enriched for immune-related function such as those involved in type I interferon signaling pathways or in negative regulation of viral genome replication. We mostly identified epigenetic signals at genes previously implicated in other SADs, for example *MX1, PARP9, DDX60*, or *IFI44L*, for which we also observed that MCTD patients exhibit higher DNA methylation variability compared with controls, suggesting that these sites might be involved in plastic immune responses that are relevant to the disease. Through methylation quantitative trait locus (meQTL) analysis we identified widespread local genetic effects influencing DNA methylation variability at MCTD-associated sites. Interestingly, for *IRF7, IFI44* genes, and the *HLA* region we have evidence that they could be exerting a genetic risk on MCTD mediated through DNA methylation changes. Comparison of MCTD-associated epigenome with patients diagnosed with SLE, or Sjögren's Syndrome, reveals a common interferon-related epigenetic signature, however we find substantial epigenetic differences when compared with patients diagnosed with rheumatoid arthritis and systemic sclerosis. Furthermore, we show that MCTD-associated CpGs are potential epigenetic biomarkers with high diagnostic value. Our study serves to reveal new genes and pathways involved in MCTD, to illustrate the important role of epigenetic modifications in MCTD pathology, in mediating the interaction between different genetic and environmental MCTD risk factors, and as potential biomarkers of SADs.

## Introduction

Mixed connective tissue disease(MCTD) is a complex rare systemic autoimmune disease (SAD) characterized by the presence of high levels of anti-U1 ribonucleoprotein (anti-RNP) autoantibodies and a mixture of signs and symptoms that resemble other systemic autoimmune diseases (SADs). MCDT was first recognized through overlapping features with systemic lupus erythematosus (SLE), systemic sclerosis (SSc), myositis, and rheumatoid arthritis (RA) (1). But its similarity to these diseases has led to controversies around its existence as a separate entity, as an overlap syndrome or as a predecessor to any one of each of the other connective tissue diseases (CTD). In fact, a small percentage of patients diagnosed with MCTD evolve into SLE or SSc (2, 3). Nowadays, MCTD is considered a disease on its own.

Since MCTD was described, four proposals for classification criteria have been published (4–6), but none of them is totally accepted by the medical community, challenging the comparison between studies (4). Currently, MCTD is diagnosed by the presence of anti-RNP antibodies, Raynaud's phenomenon, diffuse hand edema (“puffy hands”), and at least two of the following symptoms: arthritis, myositis, leukopenia, esophageal dysmotility, pleuritis, pericarditis, interstitial lung disease, or pulmonary hypertension (4). However, none of these features are unique of MCTD. Its clinical heterogeneity together with its similarities with other CTD, particularly with SSc make its diagnosis difficult. In addition, its prevalence is lower than other SADs, affecting mainly females around their 40 s. For the reasons described above, this disease has been very poorly studied at the molecular level.

As with other SADs, in the development of MCTD genetic and environmental factors combined are involved. However, its etiology is currently unknown. On the one hand, no evidence of potential environmental factors exist for MCTD (4). On the other, the role of genetics in its pathogenesis is unclear. The importance of the genes in MCTD is manifested by the presence of a family history and its comorbidity with other SADs (7). However, few genetic studies have been performed in this disease mainly due to the difficulty in obtaining large case-control cohorts, hence only HLA-DRB1^*^04:01 has been confirmed as a genetic risk factor for MCTD (8, 9).

Epigenetic modifications are defined as those structural adaptation of chromosomal regions that can register, signal, and perpetuate through cell division altered cellular activity and transcriptional states without altering DNA sequence. Epigenetic modifications can mediate individuals' immune responses to genetic risk factors as well as to various external and internal changing conditions. Their study is important for the immune system as they determine the transcriptional landscapes of cells during cell differentiation and activation of immune cells (10). The most extensively studied epigenetic mark in population-based genome-wide association studies is DNA methylation (DNAm). Alteration in the DNAm patterns has been postulated as an important cause in the development of autoimmunity (11). In the last decade, epigenome-wide association studies (EWAS) have provided insights into SADs, identifying new *loci* and pathways implicated in their pathogenesis, in the development of specific phenotypic manifestations and in the response to treatment (12). For instance, the global hypomethylation of interferon (IFN)-inducible genes is well-described and confirmed in different immune cell types of patients with SLE and Sjögren's syndrome (SjS) (12, 13). This epigenetic *interferon signature* seems to occur early in the hematopoietic process, remaining during periods of disease flares, providing a mechanism to explain type I IFN hyper-responsiveness in SLE (13).

The aim of this study is to discover novel epigenetic changes associated with MCTD, to investigate how different genetic and immune conditions can interact in shaping the variability of DNAm at MCTD-associated sites, and finally to give insight into differences between the MCTD- epigenome and other SAD pathologies. For this purpose, we performed the first epigenome-wide association study interrogating DNAm levels obtained with the newest Infinium MethylationEPIC array from whole blood samples of 31 patients diagnosed with MCTD and 255 healthy subjects and integrate our results with genetic data, clinical records and with data obtained from other SAD such as SLE, SjS, RA, and SSc.

## Results

### Genome-Wide DNA Methylation Patterns Associated With MCTD

**Figure 1:**
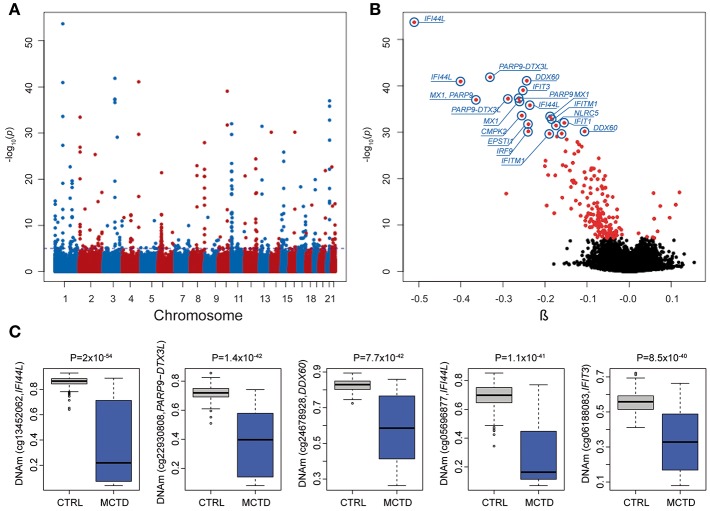
Results from epigenome-wide association study (EWAS) in mixed connective tissue disease. **(A)** Manhattan plot showing the EWAS results. *P*-values are represented on the –log10 scale in the *y-*axis. The genomic position for each CpG tested is represented in the *x-*axis. Discontinuous blue line represents the genome-wide significance threshold (*P* < 6.4 × 10^−08^). The top 10 associations are labeled with gene names. **(B)** Volcano plot of the EWAS results. *P*-values are represented on the –log10 scale in the *y-*axis. The effect size and direction of obtained in EWAS result for each CpG site is depicted in the *x-*axis. Red dots represent significant associations. The top 10 associations are labeled with gene names. **(C)** Boxplot representing the top five differentially methylated CpG-sites in cases and healthy controls. DNAm levels are presented in the y-axis. Horizontal line is the boxplot corresponds to the median DNAm levels for each group.

We explored DNAm patterns associated with MCTD using the Infinium MethylationEPIC BeadChip comparing methylation levels between 31 MCTD patients and 255 healthy controls reaching 776,283 autosomic CpG sites (see Supplementary Table 1 for demographic characteristics of the study sample). For that, we interrogated how DNAm levels at each CpG site change depending on MCTD status while correcting for age, sex, batch effects and blood cellular composition by means of a linear regression model. The observed *P*-value distribution for all association tested against the expected null distribution shows no genomic inflation (Supplementary Figure 1). In total, we observed 182 differential methylated CpGs sites (MCTD-DMS), located within 70 differentially methylated genes (MCTD-DMG) (Figure 1A, Supplementary Table 2), that passed our Bonferroni-corrected threshold for multiple testing (*P* < 6.4 × 10^−08^). Table 1 shows the results for the top 30 MCTD-DMS. In the majority of MCTD-DMS (94%), we found lower DNAm levels in patients compared with controls, supporting the observations of massive hypomethylation previously described in other related SADs (14–19) (Figure 1B). Among those MCTD-DMGs showing the largest differences in DNAm levels between patients and controls (|Δβ| > 0.2) we find genes and regions such as *IFI44L, MX1, PARP9/DTX3L, EPSTI1, IFIT3, DDX60, IFIT1*, and *NLRC5*, all of them lying within interferon-inducible genes. In an independent sample of 21 MCTD cases and 103 controls for which we had DNAm information based on 450 K bead array, we could test again 100 of these MC^20^TD-DMS (55%) and we successfully replicated 99 of them (99%, *P* < 0.05) (Supplementary Table 2). Differences in DNAm for those CpGs located within the X-chromosome were analyzed separately only in females in order to avoid sex-biased results in a total of 217 samples and 17,530 probes. At a Bonferroni-corrected significance level (*P* < 2.9 × 10^−06^) we found no evidence of differential methylation (data not shown).

**Table 1:** Top differentially methylated sites in MCTD epigenome-wide analysis.

**CpG site**	**Position**	**Gene**	**MCTDmet**	**CTRLmet**	**β**	***P***
cg13452062	1:79088559	*IFI44L*	0.35	0.86	−0.51	2.0 × 10^−54^
cg22930808	3:122281881	*PARP9; DTX3L*	0.38	0.72	−0.33	1.4 × 10^−42^
cg24678928	4:169240829	*DDX60*	0.58	0.82	−0.24	7.7 × 10^−42^
cg05696877	1:79088769	*IFI44L*	0.29	0.69	−0.40	1.1 × 10^−41^
cg06188083	10:91093005	*IFIT3*	0.35	0.55	−0.25	8.6 × 10^−40^
cg08122652	3:122281939	*PARP9; DTX3L*	0.56	0.82	−0.26	4.4 × 10^−38^
cg07815522	2:122282157	*PARP9; DTX3L*	0.44	0.72	−0.29	5.9 × 10^−38^
cg21549285	21:42799141	*MX1*	0.43	0.79	−0.36	1.0 × 10^−37^
cg00959259	3:122281975	*PARP9; DTX3L*	0.32	0.59	−0.26	2.4 × 10^−37^
cg22862003	21:42797588	*MX1*	0.41	0.64	−0.24	1.5 × 10^−36^
cg03607951	1:79085586	*IFI44L*	0.26	0.54	−0.25	2.5 × 10^−34^
cg01028142	2:7004578	*CMPK2*	0.68	0.87	−0.19	3.6 × 10^−34^
cg26312951	21:42797847	*MX1*	0.21	0.37	−0.18	1.6 × 10^−33^
cg03038262	11:315262	*IFITM1*	0.41	0.52	−0.15	9.6 × 10^−33^
cg05552874	10:91153143	*IFIT1*	0.41	0.67	−0.24	1.8 × 10^−32^
cg06562969	13:43567153	*EPSTI1*	0.49	0.63	−0.17	3.5 × 10^−32^
cg07839457	16:57023022	*NLRC5*	0.26	0.49	−0.24	6.4 × 10^−31^
cg25998594	14:24632095	*IRF9*	0.78	0.87	−0.11	6.7 × 10^−31^
cg05883128	4:169239131	*DDX60*	0.37	0.50	−0.16	1.9 × 10^−30^
cg23570810	11:315102	*IFITM1*	0.45	0.60	−0.19	1.9 × 10^−30^
cg06981309	3:146260954	*PLSCR1*	0.26	0.46	−0.17	8.0 × 10^−30^
cg13155430	21:42795929	*MX1*	0.75	0.90	−0.15	3.5 × 10^−29^
cg21995613	8:144106922	*Intergenic*	0.65	0.75	−0.12	1.2 × 10^−28^
cg13304609	1:79085162	*IFI44L*	0.73	0.85	−0.12	4.5 × 10^−28^
cg14595557	2:7006786	*CMPK2*	0.29	0.42	−0.11	1.2 × 10^−27^
cg10959651	2:7018020	*RSAD2*	0.14	0.26	−0.12	1.3 × 10^−26^
cg05475649	15:45007015	*B2M*	0.43	0.53	−0.15	1.4 × 10^−26^
cg08888522	2:163172908	*IFIH1*	0.73	0.87	−0.13	4.4 × 10^−26^
cg04880620	12:113415945	*OAS2*	0.46	0.51	−0.07	4.0 × 10^−25^
cg27537252	15:45006400	*B2M*	0.32	0.48	−0.20	1.3 × 10^−24^

*MCTDmet represents mean methylation level in MCTD cases*.

*CTRLmet represents mean methylation level in controls*.

*β represents DNAm difference between controls and MCTD cases*.

*P is the P-value obtained in the linear regression model adjusted by age, sex, batch effects, and estimated cell proportions*.

*Genomic positions are based on the hg19 human reference sequence build (GRCh37)*.

We next performed a genome-wide search for CpG-sites showing differences in DNAm variability across MCTD patients and controls. We called these sites as MCTD-associated variable methylated sites (MCTD-VMS). In total, we observed 97 MCTD-VMS, located within 50 variable methylated genes (MCTD-VMG) (Supplementary Table 3) that passed our Bonferroni-corrected threshold for multiple testing (*P* < 6.4 × 10^−08^). Table 2 shows results for the top 30 MCTD-VMS. For every MCTD-VMS we found higher DNAm variability in MCTD patients compared with controls. MCTD-VMS could represent CpG sites with altered and plastic DNAm profiles underlying different pathological molecular processes. In our replication sample we could test 53 of these MCTD-VMS (54%) and we successfully replicated 40 of them (75%, *P* < 0.05) (Supplementary Table 3). We found an overlap of 83 MCTD-VMS with MCTD-DMS, indicating that a large fraction of differentially methylated sites associated with MCTD also show increased variability. Figure 1C shows DNAm variability across MCTD and controls for the top 5 associated DMS.

**Table 2:** Top variable methylated sites in MCTD epigenome-wide analysis.

**CpG site**	**Position**	**Gene**	**MCTDvar**	**CTRLvar**	**LevTest**	***P***
cg01028142	2:7004578	*CMPK2*	0.172	0.015	223.8	1.0 × 10^−37^
cg21549285	21:42799141	*MX1*	0.287	0.053	217.9	5.4 × 10^−37^
cg08122652	2:122281939	*PARP9; DTX3L*	0.214	0.033	216.4	8.3 × 10^−37^
cg24678928	4:169240829	*DDX60*	0.186	0.033	195.8	3.4 × 10^−34^
cg07815522	3:122282157	*PARP9; DTX3L*	0.227	0.042	192.4	9.4 × 10^−34^
cg08888522	2:163172908	*IFIH1*	0.141	0.023	186.4	5.6 × 10^−33^
cg13304609	1:79085162	*IFI44L*	0.125	0.035	184.9	8.9 × 10^−33^
cg24298610	21:42796936	*MX1*	0.090	0.025	179.9	4.1 × 10^−32^
cg22930808	3:122281881	*PARP9; DTX3L*	0.236	0.045	174.9	1.9 × 10^−31^
cg05883128	4:169239131	*DDX60*	0.151	0.041	169.2	1.1 × 10^−30^
cg08926253	11:614761	*IRF7*	0.131	0.040	165.1	4.3 × 10^−30^
cg13155430	21:42795929	*MX1*	0.146	0.021	162.8	8.8 × 10^−30^
cg22862003	21:42797588	*MX1*	0.180	0.046	160.5	1.8 × 10^−29^
cg06708931	8:144103857	*Intergenic*	0.100	0.015	145.7	2.3 × 10^−27^
cg25998594	14:24632095	*IRF9*	0.097	0.023	145.6	2.4 × 10^−27^
cg06033320	8:66750110	*PDE7A*	0.165	0.066	134.6	9.7 × 10^−26^
cg12037516	11:614954	*IRF7*	0.075	0.022	130.5	4.0 × 10^−25^
cg05552874	10:91153143	*IFIT1*	0.180	0.054	119.4	1.9 × 10^−23^
cg03038262	11:315262	*IFITM1*	0.140	0.057	119.2	2.1 × 10^−23^
cg01079652	1:79118191	*IFI44*	0.177	0.034	117.3	4.2 × 10^−23^
cg22016995	11:614787	*IRF7*	0.114	0.012	117.2	4.2 × 10^−23^
cg21995613	8:144106922	*Intergenic*	0.121	0.047	116.1	6.3 × 10^−23^
cg12906975	8:144105259	*Intergenic*	0.066	0.012	108.9	8.5 × 10^−22^
cg13452062	1:79088559	*IFI44L*	0.327	0.040	108.9	8.5 × 10^−22^
cg16400320	8:144105210	*Intergenic*	0.063	0.019	108.2	1.1 × 10^−21^
cg11702942	8:144102584	*LY6E*	0.089	0.034	106.7	1.9 × 10^−21^
cg25984164	1:174844560	*RABGAP1L*	0.142	0.065	104.3	4.5 × 10^−21^
cg14293575	22:18635460	*USP18*	0.184	0.028	97.0	7.1 × 10^−20^
cg14864167	8:66751182	*PDE7A*	0.200	0.073	96.2	9.6 × 10^−20^

*MCTDvar represents DNA methylation variability in MCTD cases measured as its standard deviation*.

*CTRLvar represents DNA methylation variability in controls measured as its standard deviation*.

*LevTest represents the test statistic obtained in the Levene's test*.

*P is the P value obtained for Levene's test comparing variance between MCTD cases and controls in DNAm residuals after adjusted by age, sex, batch effects, and estimated cell proportions*.

*Genomic positions are based on the hg19 human reference sequence build (GRCh37)*.

To identify common functional characteristics of MCTD-associated epigenetic alterations, we performed gene ontology (GO) analysis of biological processes including all differentially methylated genes. Functional enrichment analysis revealed that type I interferon signaling pathway (GO:0060337), cellular response to type I interferon (GO:0071357), and interferon-gamma-mediated signaling pathway (GO:0060333) were the most significantly enriched GO terms (adjusted *P* < 7 × 10^−19^). Other immune-related functions related to the innate immunity such as cytokine-mediated signaling pathway (GO:0019221), negative regulation of viral life cycle (GO:1903901) and negative regulation of viral genome replication (GO:0045071) were among the top significant GO terms (adjusted *P* < 7 × 10^−15^) (Supplementary Table 4).

### Treatment Effects on MCTD-Associated Epigenetic Signals

**Figure 2:**
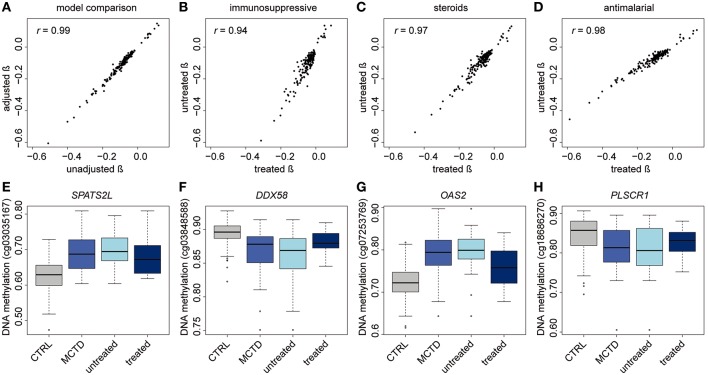
Treatment effects on the MCTD epigenetic associations. **(A–D)** Effect size correlation plots to compare results from different models. **(A)** In the *x-*axis results from linear models in which the treatments are not included as covariates are depicted. In the *y-*axis results from linear models in which treatments are included as covariates are depicted. **(B–D)** Effect size comparisons for treatment-stratified analyses comparing effect sizes in the treated sample and in the untreated sample. **(E–H)** Boxplots for immunosuppressive specific epigenetic signals. DNAm levels are illustrated in controls, MCTD cases, untreated MCTD cases and treated MCTD cases in different CpG sites.

DNAm levels fluctuate when exposed to environmental exposures such as the intake of drugs (20). MCTD patients often receive one or more treatments throughout their lives to diminish disease symptoms. At the time of blood sampling, MCTD patients included in this study were mostly exposed to therapies such as steroids, antimalarials and/or immunosuppressants (Supplementary Table 1). To investigate the influence of this exposure on our findings, we first explored whether or not DNAm levels at MCTD-DMS were still associated with MCTD diagnosis when treatments are included as covariates in the linear model. All previously described MCTD-DMS remained significantly associated after treatment correction at least at a significance level as low as *P* < 0.007 (Supplementary Table 2), moreover we observed a high correlation between the effects of the disease on DNAm levels and the significance level obtained from the model with and without treatment correction (Pearson's *r* > 0.95, *P* < 2.2e−16) (Figure 2A), which suggests that the MCTD epigenetic signature is not fully explained or significantly influenced by therapy.

In order to further explore whether or not there exists DNAm patterns associated with the disease that are dependent on specific treatments, we stratified the MCTD patients according to treatment and compared the results obtained between the untreated and the treated group (Figures 2B–D). For example, when stratifying the associations by immunosuppressive treatment, we observed a high correlation (Pearson's *r* = 0.94) between effect sizes obtained in the untreated and treated groups, indicating that the genome-wide epigenetic signature does not depend on the therapy. However, we observed a tendency of effect sizes being generally larger in the untreated group than in the treated, which indicates that the epigenetic state of those individuals under immunosuppressive treatment resembles more that of the healthy population (Figure 2B). The same is seen for those patients under steroid therapy (Figure 2C). Interestingly, an opposite pattern is observed for the group using antimalarials whose epigenetic profile differs more from the untreated group (Figure 2D). Finally, we individually searched for treatment-specific effects among MCTD-DMS by selecting those associations that were significant in one group (after correction for multiple testing) but with little evidence for association in the other (*P* > 0.01) (Supplementary Tables 5, 6). Due to our limited sample size in the stratified analyses these results are likely to be influenced by the little power, we only report as treatment-specific effects those that could be replicated in our independent sample. We found robust immunosuppressive-specific epigenetic effects at the genes *PARP14, SPATS2L, DDX58, OAS2, PLSCR1, HLA-F, LGALS3B* (Table 3). Examples for these treatment-specific effects are exemplified in Figures 2E–H in which DNAm levels are depicted in the healthy group, in all MCTD patients, in those untreated subjects, and in the treated group for which we can see a reversal of DNAm levels toward the control group. Interestingly, for *SPATS2L* and *OAS2* genes, we can see how MCTD patients exhibit an hypermethylation compared with controls, as well as the treated subjects compared with the untreated ones, which is the opposite trend for what is seen in most MCTD-DMS. Our results indicate that some epigenetic associations are, at least partially, modified by therapy, and suggest that targeting specific-epigenetic alterations could be a potential molecular mechanism to revert the disease.

**Table 3:** Treatment stratification epigenetic association results for immunosuppressive therapy.

			**Discovery sample (EPIC)**		**Replication sample (450K)**	
**CpG site**	**Position**	**Gene**	**Treated β (P)**	**Untreated β (P)**	**Treated β (P)**	**Untreated β (*P*)**
cg01721555	3:122401300	*PARP14*	−0.01 (0.072)	−0.03 (4.5 × 10^−12^)	−0.01 (0.208)	−0.04 (2.0 × 10^−04^)
cg03035167	2:201336269	*SPATS2L*	0.01 (0.238)	0.05 (6.9 × 10^−10^)	0.02 (0.019)	0.05 (3.0 × 10^−06^)
cg03848588	9:32525008	*DDX58*	−0.01 (0.149)	−0.04 (4.4 × 10^−15^)	−0.01 (0.043)	−0.03 (1.8 × 10^−04^)
cg07253769	12:113447342	*OAS2*	0.03 (0.014)	0.07 (4.2 × 10^−12^)	0.02 (0.171)	0.06 (4.3 × 10^−04^)
cg18686270	3:146258875	*PLSCR1*	−0.01 (0.177)	−0.04 (4.4 × 10^−11^)	−0.01 (0.396)	−0.07 (5.9 × 10^−04^)
cg23892836	6:29692085	*HLA-F*	−0.04 (0.016)	−0.07 (2.6 × 10^−10^)	−0.04 (0.012)	−0.07 (4.6 × 10^−04^)
cg25178683	17:76976267	*LGALS3BP*	−0.02 (0.198)	−0.11 (3.6 × 10^−16^)	−0.04 (0.026)	−0.07 (5.3 × 10^−04^)

*Treated β (P) represents the DNA methylation differences between treated MCTD cases and controls*.

*Untreated β (P) represents the DNA methylation differences between untreated MCTD cases and controls*.

*P represents the P-value obtained from in the linear regression model adjusted by age, sex, batch effects, and estimated cell proportions*.

*Genomic positions are based on the hg19 human reference sequence build (GRCh37)*.

### Genetic Drivers of MCTD-Associated Differential Methylation

Other than environmental and stochastic factors, genetic variation is also an important contributor in shaping DNAm levels across the genome, as described in several recent genetic DNAm quantitative trait loci (meQTL) studies (20–23). In order to gain insight into the genetic control of the MCTD-associated epigenetic signals, we performed *cis*-meQTL analyses to detect genetic variants located closely to MCTD-DMS (no further than 1 Mb) that influence DNAm irrespectively of disease status. For that, we included 259 subjects (29 MCTD and 230 CTRL) for which both EPIC DNAm and genetic data were available. The observed *P*-value distribution for all cpg-SNP associations tested against the expected null distribution shows no genomic inflation and is depicted in Supplementary Figure 2. At a False Discovery Rate (FDR) < 0.05 we detected 2,077 genetic-epigenetic associations involving 31 MCTD-DMS and 1,806 SNPs, many of which are in high linkage disequilibrium (LD) (Supplementary Table 7). MCTD-associated *cis*-meQTLs are distributed across 21 differentially methylated genes, or MCTD-DMGs, among which *EPST1, VRK2, ADAR*, and *IRF7* are the genes whose DNAm pattern show the strongest genetic control. Table 4 shows the top genetic effects for each MCTD-DMG. In an independent sample of 20 MCTD cases and 96 controls for which we had DNAm information based on 450 K bead array, we could test again 909 of these MCTD-DMS (37%) sitting along 25 genes, and we successfully replicated meQTLs for 11 MCTD-DMGs (40%, at a significance level of *P* < 0.001) (Supplementary Table 8). These results demonstrate that there is a widespread effect of local genetic variants on MCTD-associated DNAm sites.

**Table 4:** Best meQTL results per MCTD-DMS.

**CpG site**	**CpG position**	**CpG gene**	**SNP**	**Alleles**	**SNP position**	**SNP gene**	**β**	***P***
cg12439472	13:43565399	*EPSTI1*	rs4142312	GA	13:43574986	*EPSTI1*	−0.167	2.6 × 10^−44^
cg09858955	2:58135951	*VRK2*	rs2678900	GT	2:58177683	*VRK2*	0.056	3.3 × 10^−20^
cg07878065	18:2641871	*Intergenic*	rs9958281	AG	18:2641809	*Intergenic*	0.019	1.1 × 10^−19^
cg03879629	3:46152446	*Intergenic*	rs13085367	CT	3:46172824	*Intergenic*	0.072	2.7 × 10^−18^
cg04268125	1:154579384	*ADAR*	rs9616	TA	1:154555733	*ADAR*	0.060	7.9 × 10^−18^
cg23892836	6:29692085	*HLA-F*	rs2517910	AC	6:29688501	*HLA-F*	−0.030	4.8 × 10^−16^
cg17114584	11:613792	*IRF7*	rs3740648	TG	11:596672	*PHRF1*	−0.084	4.0 × 10^−15^
cg00272009	3:122398855	*PARP14*	rs2668339	CG	3:122392433	*PARP14*	−0.040	1.1 × 10^−14^
cg23923934	6:31322914	*HLA-B*	rs1051488	TC	6:31322911	*HLA-B*	−0.039	1.3 × 10^−14^
cg12331471	2:231282369	*SP100*	rs12694859	TC	2:231254570	*SP140L*	−0.029	9.2 × 10^−14^
cg06981309	3:146260954	*PLSCR1*	rs2738918	CT	3:146257165	*PLSCR1*	0.039	1.1 × 10^−12^
cg14864167	8:66751182	*PDE7A*	rs6472232	GT	8:66792632	*PDE7A*	−0.048	1.7 × 10^−12^
cg12110437	8:144098888	*LY6E*	rs7812819	GA	8:144095249	*RP11-273G15.2*.	−0.046	1.0 × 10^−11^
cg23352030	20:62198469	*PRIC285*	rs9784182	GA	20:62263201	*Intergenic*	0.054	1.0 × 10^−11^
cg13755924	21:42791937	*MX1*	rs78554586	AG	21:42808343	*MX1*	−0.053	1.9 × 10^−11^
cg14880222	1:79143979	*Intergenic*	rs7524036	AC	1:79158657	*Intergenic*	0.029	1.0 × 10^−10^
cg08293824	3:172313318	*Intergenic*	rs234055	GT	3:172310820	*RP11-408H1.3*	0.038	1.8 × 10^−10^
cg07839313	19:17514600	*BST2*	rs12971834	TC	19:17516689	*CTD-2521M24.9,/BST2*	−0.027	3.8 × 10^−10^
cg06033320	8:66750110	*PDE7A*	rs6472232	GT	8:66792632	*PDE7A*	−0.034	5.4 × 10^−10^
cg07596065	22:50984393	*Intergenic*	rs9306547	CT	22:50993225	*SYCE3*	0.016	8.4 × 10^−10^
cg12013713	7:139760671	*PARP12*	rs7805521	AT	7:139761176	*PARP12*	0.032	7.9 × 10^−09^
cg09379489	12:12224360	*BCL2L14*	rs4763773	GA	12:12225613	*BCL2L14*	−0.024	1.6 × 10^−08^
cg24603130	18:60253492	*ZCCHC2*	rs57868717	TC	18:60247316	*ZCCHC2*	−0.032	2.3 × 10^−08^
cg16427501	8:145060083	*PARP10*	rs11136343	GA	8:145058986	*PARP10*	0.017	4.2 × 10^−08^
cg26202327	2:233193151	*DIS3L2*	rs10933388	AT	2:233176366	*DIS3L2*	−0.035	5.0 × 10^−08^
cg10734665	15:26107410	*ATP10A*	rs12908995	AG	15:26015194	*ATP10A*	−0.025	7.5 × 10^−08^

*Alleles represents the allele tested in first place followed by the non-tested allele for each SNP*.

*β represents the DNA methylation change in the addition of one allele tested*.

*P represents the P-value obtained from the linear regression model adjusted by age, sex, batch effects, estimated cell proportions, disease status and first genetic component*.

*Genomic positions are based on the hg19 human reference sequence build (GRCh37)*.

### Intermediary Role of DNA Methylation in MCTD Genetic Risk

Next, we addressed the question of whether or not SNPs involved in meQTL do show differences in allele frequencies between patients and controls and could represent MCTD-associated genetic variants exerting their genetic risk on the disease through DNAm changes. For that, we performed genetic association testing by means of logistic regression analyses in an extended sample of 89 MCTD and 550 CTRLs for which we had genotypic data. At a suggestive significance level of *P* < 0.05, we found 168 genetic variants that are associated both with DNAm levels at 8 CpG sites and show some evidence of being associated with risk for MCTD, that could potentially impact on MCTD pathology by changing DNAm levels in a *cis*-regulating manner, many of them sitting in the same haplotypic block and influencing the same CpG site. (Supplementary Table 9). Table 5 shows the top MCTD-associated SNPs influencing DNAm levels at MCTD-DMS. To give further support to the robustness of their associations and their link with autoimmunity, we investigated if they are also genetically associated with other immune-related diseases such as SLE, SjS, RA, and SSc for which we also had available genotypic data. Importantly, genetic variants at *IFI44* (rs1051047), the intergenic region at chr18 (rs73936737), and the HLA-region (rs2251892, rs3130251) gave convincing evidence of their genetic association with SLE and/or SjS (0.05 > *P* > 4 × 10^−08^) (Table 5, Supplementary Table 9).

**Table 5:** Genetic effects of MCTD-meQTLs in MCTD and other related SADs.

**CpG site**	**CpG location**	**CpG Gene**	**SNP**	**SNP location**	**SNP Gene**	**Alleles**	**AF**	**meQTL**	**EWAS β (P)**	**MCTD OR (P)**	**SAD (OR,P)**
							**MCTD/CTRL**	**β (P)**			
cg15331332	chr6:29692111	*HLA-F*	rs3130251	chr6:29629344	*MOG*	CT	0.24/0.15	−0.03	−0.06	1.86 (0.0014)	SLE(1.63, 2 x 10^−05^)
								(9.8 x 10^−08^)	(2.9 x 10^−08^)		SjS (1.94, 4 x 10^−08^)
cg23892836	chr6:29692085	*HLA-F*	rs2251892	chr6:29833128	*HLA-H/HLA-G*	TC	0.43/0.33	−0.02	−0.06	1.59 (0.004)	SLE (1.23,0.03)
								(8.8 x 10^−09^)	(1.4 x 10^−09^)		SjS (1.53, 2 x 10^−05^)
cg17114584	chr11:613792	*IRF7*	rs3740648	chr11:596672	*PHRF1*	TG	0.14/0.08	−0.08	−0.14	1.98 (0.007)	–
								(4.0 × 10^−15^)	(1.7 × 10^−22^)		
cg07878065	chr18:2641871	intergenic	rs73936737	chr18:2635566	intergenic (chr18)	GA	0.15/0.26	0.01	−0.03	0.57 (0.007)	SLE (0.75, 0.007)
								(9.9 × 10^−08^)	(5.0 × 10^−09^)		
cg14880222	chr1:79143979	intergenic	rs1051047	chr1:79129694	*IFI44*	GA	0.50/0.60	0.03	−0.04	0.40 (0.017)	SLE (0.72, 0.05),
								(1.7 × 10^−09^)	(4.3 × 10^−09^)		SjS (0.72, 0.06)
cg04268125	chr1:154579384	*ADAR*	rs11264235	chr1:154631081	intergenic (chr1)	TC	0.40/0.28	−0.04	−0.2	1.43 (0.036)	–
								(4.7 × 10^−08^)	(2.0 × 10^−23^)		
cg00272009	chr3:122398855	*PARP14*	rs16834903	chr3:122379614	intergenic (chr3)	AG	0.40/0.36	−0.04	−0.09	1.37 (0.048)	–
								(5.7 × 10^−14^)	(1.9 × 10^−11^)		
cg26882438	chr3:122399120	*PARP14*	rs16834903	chr3:122379614	intergenic (chr3)	AG	0.40/0.36	−0.03	−0.14	1.37 (0.048)	–
								(1.7 × 10^−10^)	(2.6 × 10^−20^)		

*Alleles represent the allele tested in first place followed by the non-tested allele for each SNP. Tested allele frequency is given in parenthesis*.

*AF corresponds to the allele frequency of the tested allele in cases and controls*.

*meQTL β (P) represents the DNAm change in the addition of one tested allele together with the corresponding P value from the linear regression model adjusted by age, sex, batch effects, estimated cell proportions, disease status and first genetic component*.

*EWAS β (P) represents DNAm difference between controls and MCTD cases from epigenome-wide association study together with the P value obtained in the linear regression model adjusted by age, sex, batch effects and estimated cell proportions*.

*MCTD (OR,P) represents the Odd Ratio obtained from genetic association testing based on logistic regression model adjusted by age, sex, batch effects, estimated cell proportions and first genetic component and its corresponding P value*.

*SAD (OR, P) represents the odd ratio and P value obtained for other diseases*.

*Genomic positions are based on the hg19 human reference sequence build (GRCh37)*.

**Figure 3:**
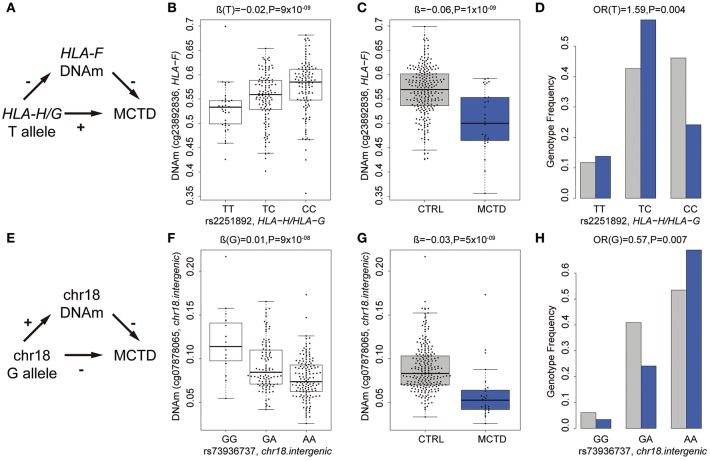
Intermediary role of DNAm in the genetic risk of MCTD. **(A)** Summary of relationships between *PHRF1*-genotype (rs12362352), DNAm at *IRF7* (cg17114584), and MCTD. The *p*-values shown were obtained in the corresponding meQTL, EWAS, or genetic association analyses. **(B)** G minor allele for *PHRF1*-genotype (rs12362352) is associated with a decreased in DNAm at *IRF7* gene (cg17114584). **(C)** DNAm at DNAm at *IRF7* gene (cg17114584) shows decreases DNAm levels in MCTD patients than controls (CTRL). **(D)** G minor allele for *PHRF1*-genotype (rs12362352) is associated with increased MCTD risk. **(E)** Summary of relationships between *HLA-B*-genotype (rs2507991), DNAm at *HLA-B* (cg23923934), and MCTD. **(F)** A minor allele for *HLA-B*-genotype (rs2507991) is associated with an increased in DNAm at *HLA-B* gene (cg23923934). **(G)** DNAm at *HLA-B* gene (cg23923934) shows decreases DNAm levels in MCTD patients than controls (CTRL). **(H)** A minor allele for *HLA-B*-genotype (rs2507991) is associated with decreased MCTD risk.

As an example of the potential intermediary role of DNm in MCTD pathology, we illustrate in Figures 3A–F the relationship between genetic variants, DNAm and MCTD for an intergenic region in chromosome 18 and for the *HLA-F* gene. The presence of the T minor allele (rs2251892) in the *HLA-G* and *HLA-H* region in chromosome 6 is associated with a decrease in DNAm levels (cg23892836) at the upstream located *HLA-F* gene (Figure 3B) and with higher MCTD risk (Figure 3D). Such relationships support the scenario in which this risk variant, which is found at higher frequencies in MCTD patients (Figure 3D), is implicated in the disease by reducing DNAm levels at this HLA class I histocompatibility antigen (Figures 3A,B). On the contrary, the presence of the G minor allele (rs73936737) in the chromosome 18 intergenic region, is linked with an increase in DNAm nearby and associated with a lower MCTD risk (Figures 3F–H). This indicates that the genetic variant could exert its protective role by reducing DNAm levels at the intergenic region (Figure 3E). These results demonstrate that MCTD-specific differences in DNAm may be driven by underlying genetic variants, and provide a molecular mechanism by which genetic polymorphisms can contribute to disease.

### Epigenetic Sharing Across Other Systemic Autoimmune Diseases

**Figure 4:**
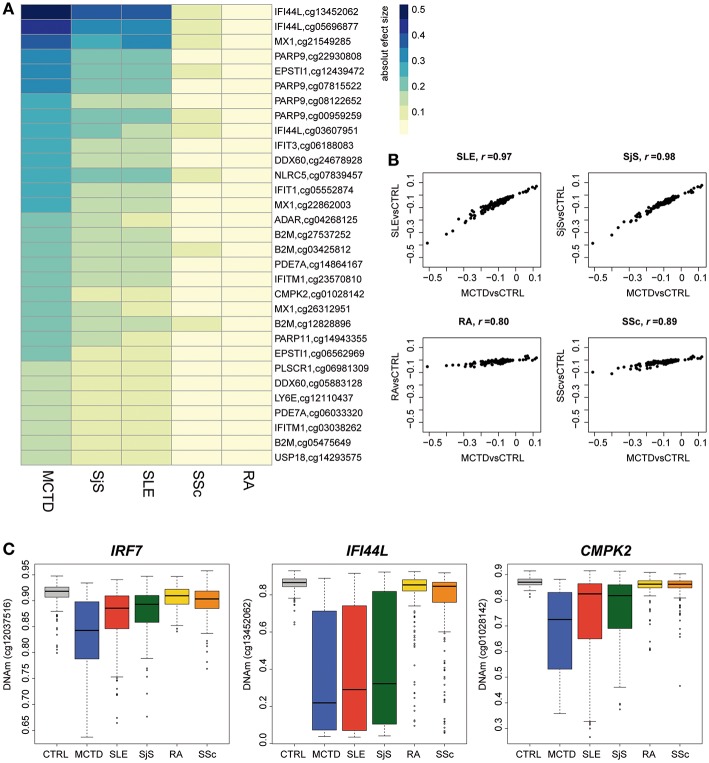
Epigenetic patterns of MCTD-epigenetic signatures in other related systemic autoimmune diseases. **(A)** Heatmap representing the absolute effect magnitude of epigenetic associations for different systemic autoimmune diseases at those MCTD-DMS showing absolute effect sizes higher than 0.15. Each column represents one disease, each row represents the absolute effect size obtained for the corresponding disease when compared with healthy subjects. **(B)** Correlation plots comparing the effect sizes obtained in the MCTD epigenetic association results (in the *y-*axis) with those obtained for other systemic autoimmune diseases (*x-*axis). **(C)** Boxplots representing the top differentially methylated CpG-sites between MCTD cases and other SADs. DNAm levels are illustrated in controls, MCTD, SLE, SjS, RA, and SSc.

MCTD is a SAD that exhibits a high degree of sharing of symptoms and serology with other SADs making it difficult to diagnose and treat. Moreover, recent EWAS on other SADs, such as SjS and SLE, have revealed a common epigenetic signature at IFN-inducible genes that we also observe in the blood of MCTD patients (14–17, 19, 24). Here, we explored to what extent the MCTD-associated epigenetic signals are distinguishable from those of other related SADs, such as SLE, SjS, RA, and SSc. For that, we used 234, 206, 217, and 177 samples, respectively. We first investigated whether or not MCTD-DMS are also differentially methylated in other SADs when compared with the healthy population. To illustrate the results of this cross-diseases comparison, we have represented in a heatmap the effect sizes obtained for each disease and for 31 CpG-sites for which we observed an absolute DNAm difference higher than 0.15 when compared with controls (Figure 4A). At a Bonferroni-significance level of *P* < 2.7 × 10^−04^, we observed that most MCTD-associated epigenetic sites are also SLE-DMS (98%) and SjS-DMS (98%), while a much lower fraction are found to be associated with SSc (20%) or with RA (15%) (Supplementary Table 10). Indeed, when comparing the magnitude of the effects of MCTD-epigenetic associations with those from different diseases, we found the highest effect sizes correlation between MCTD and SjS models (Pearson's *r* = 0.98) followed by SLE (Pearson's *r* = 0.97), and a substantially lower correlation with SSc (Pearson's *r* = 0.89) and RA (Pearson's *r* = 0.80), and hence reduced similarities with these effect sizes (Figure 4B). This pattern was consistent in our replication sample (Supplementary Figure 3). Interestingly, we observed that the difference between DNAm levels is usually higher for MCTD than for SjS and/or SLE when compared with the healthy population (Figure 4A, Supplementary Table 10).

Finally, we interrogated whether or not there exist significant DNAm differences between MCTD and every other disease at the entire set of MCTD-DMS (Supplementary Table 11). At a Bonferroni-significance level of *P* < 2.7 × 10^−04^, we observed no significant DNAm difference between MCTD and SLE, while only 1 CpG showed significant differences in DNAm between MCTD and SjS. However, we detected a large number of differential methylation sites for RA and SSc (154 and 137, respectively). Figure 4C shows the DNAm profile across the different diseases and the healthy population for the top differentially methylated genes. CpG cg12037516 (*IRF7* gene) showed the largest DNAm difference between MCTD and SjS patients. CpG cg13452062 (*IFI44L* gene) showed the largest DNAm difference between MCTD and RA patients. And finally, the CpG cg01028142 (*CMPK2* gene) showed the largest DNAm difference between MCTD and SSc.

Our results reveal in first place that MCTD is epigenetically linked to SLE and SjS, as they all shared a common IFN epigenetic signature, but that for MCTD a more striking pattern of DNAm differentiation is observed. We also show that there exist substantial epigenetic differences with RA and SSc, which indicates that these epigenetic dissimilarities can be of interest to develop new blood biomarkers that could distinguish effectively MCTD from other SADs, as it is explored in the next section.

### Diagnostic Utility of MCTD-Associated Methylation to Differentiate MCTD From Healthy Populations and Other Autoimmune Systemic Diseases

**Figure 5:**
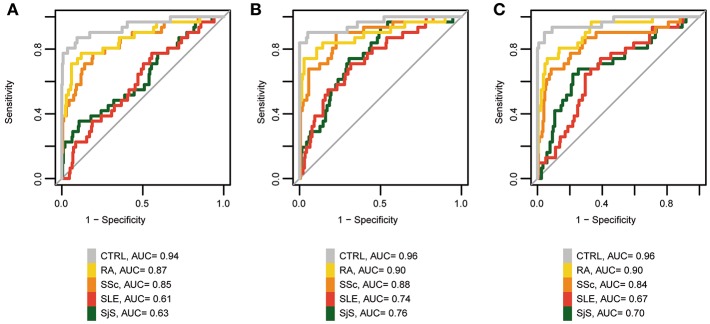
Diagnostic value of MCTD-associated epigenetic signals. **(A)** ROC curves for *PARP9* DNAm (cg22930808) to discriminate patients with MCTD from the healthy population (CTRL), SLE, SjS, RA, and SSc. **(B)** ROC curves for the top 10 CpG-sites discriminating MCTD from CTRLs (cg22930808, cg03607951, cg13452062, cg22862003, cg06981309, cg05552874, cg07839457, cg24678928, cg00959259, cg05696877) to discriminate patients with MCTD from the healthy population (CTRL), SLE, SjS, RA, and SSc. **(C)** ROC curves of *IFI44L* DNAm. (cg13452062, cg05696877, cg03607951, cg13304609, cg17980508, cg00458211) to discriminate patients with MCTD from healthy population (CTRL), SLE, SjS, RA, and SSc. AUC, Area under the curve.

Lastly, we wanted to evaluate the diagnostic value of MCTD-associated epigenetic markers to distinguish MCTD patients from healthy population, but also from the other 4 distinct systemic autoimmune related diseases (SLE, SjS, RA, and SSc). Firstly, we conducted logistic regression and ROC curves analyses based on DNAm levels at individual CpG sites for the 31 MCTD-DMS that show the highest DNAm differences (|Δβ| > 0.15) between MCTD and healthy subjects. DNAm status at 10 CpGs sitting in genes *PARP9, IFI44L, MX1, PLSCR1, IFIT1, NLRC5*, and *DDX60* show high diagnostic performance to distinguish MCTD from healthy patients (Area Under Curve (AUC) > 0.90), being the highest cg22930808 at *PARP9* gene which exhibited an AUC of 0.94 (Figure 5A, Supplementary Table 12). We found no CpG that individually could distinguish proficiently MCTD patients from those diagnosed with SLE or SjS (AUC < 0.65). However, we found a good diagnostic value (AUC > 0.80) to discriminate MCTD from RA and SSc at 17 and 10 CpG sites, respectively, being the highest cg13452062 at *IFI44L* for RA (AUC = 0.87) and cg22930808 at *PARP9* gene for SSc (AUC = 0.85). Importantly, when we added the information of DNAm at the top 10 discriminating CpG sites in the logistic regression as predicting covariates, we increased our MCTD diagnostic ability reaching AUC values as high as 0.96 to differentiate from the healthy population, 0.90 to differentiate from RA patients and 0,88 to differentiate from SSc (Figure 5B). Notably, we also obtained a reasonable good performance to discriminate MCTD from SLE and SjS (AUC = 0.76 and 0.74, respectively), which surprisingly makes epigenetic biomarkers a very interesting tool to distinguish between epigenetically resembling diseases as well (Figure 5B).

Lastly, DNAm levels at *IFI44L* gene has been previously described to serve as an effective biomarker to distinguish SLE from the healthy population, as well as from SjS and RA (25). Here we interrogated the diagnostic utility of DNAm to differentiate MCTD between the healthy population and other SADs-related diseases. For that, we evaluated the joined diagnostic value of 6 *IFI44L*-MCTD-DMS (cg13452062, cg05696877, cg03607951, cg13304609, cg17980508, and cg00458211). Our results show a very high *IFI44L* discriminating value to differentiate MCTD between the healthy population, RA and SSc patients (AUC = 0.97, 0.90, and 0.84, respectively), but not as good ability to discriminate MCTD from SLE and SjS patients (AUC < 0.71), as when putting the epigenetic information from different interferon-related genes together (Figures 5B,C). These results were highly concordant with those obtained in our replication independent sample based on 450 K data (Supplementary Table 13), which serves to replicate and/or cross-validate our results and to give robustness to these findings.

## Discussion

This is the first time that MCTD is studied at the molecular, and particularly at the epigenetic level. We have found widespread DNAm changes associated with MCTD, the vast majority of them shows decreased DNAm levels in MCTD patients compared with healthy subjects in genes that are transcriptionally responsive to the presence of interferon or involved in type I interferon pathways. Most of our signals are located in genes that have been previously reported in EWAS studies to be associated with other related SADs, such as SLE and SjS in whole blood, but also in other different fractioned blood cell types (14–17, 19, 24). However, this is it the first time, to be best of our knowledge, that the epigenetic state of an autoimmune disease is interrogated at the resolution of > 700,000 CpG sites in the genome (Infinium MethylationEPIC array). Functional enrichment analysis based on EPIC methylation data confirm the epigenetic IFN signature and expands the list of CpG-associated with autoimmune diseases. Our study also reveals that for most MCTD-associated epigenetic signals, an increase in DNAm variability is observed. This disease-associated increased DNAm variability has been previously seen for other immune-related conditions and could reflect different scenarios (19, 26, 27). Firstly, it could be the result of the plastic nature of the activated immune cells involved in the disease (28). Secondly, increases in DNAm variability can underlie the contribution of many different molecular processes to MCTD etiology, which could be reflected as well in the high heterogeneity observed at the level of clinical manifestations, as it is also the case for other SADs.

The MCTD patients involved in this study were mostly under steroids, antimalarial or immunosuppressive medication at the time of blood sampling. We show that adjusting our analyses for treatment did not change significantly our results, which indicates that our findings are not mainly driven by treatment effects. However, we see a general trend toward a more pronounced DNAm difference in the untreated group and intermediate DNAm levels in the treated group compared with healthy subjects. Indeed, a number of CpG sites, for example those in genes such as *OAS2, PLSCR1, SPATS2L*, and *DDX58*, could only be detected as significantly associated with MCTD in the untreated group. These results were largely confirmed in our replication sample and suggest that at least some epigenetic associations are modified by therapy. These findings open a gate to study how targeting the epigenetic state at IFN-related genes could be a potential molecular drug mechanism to revert the disease.

A number of previous studies have suggested that DNAm could be a mediator of genetic risk in disease (19, 21, 29), which provides a functional mechanism to link genetic variation with disease. By integrating genetic and DNAm data we conducted meQTL analyses and observed strong evidence of *cis* genetic regulation of DNAm levels at a large fraction of MCTD-DMS. To date, there is no genome-wide association study published for MCTD. The fact that MCTD is a rare complex disease exhibiting a mixture of heterogeneous symptoms that resembles other SADs has challenged the recruitment of large enough samples to conduct GWAS studies. However, here we could explore whether genetic variants involved in MCTD-meQTLs do show some evidence of being genetically associated with MCTD and/or with other related SADs. Interestingly, we reveal that genetic variants at 5 loci are both associated with MCTD risk and with DNAm levels at genes *IFI44, PHRF1*, in the HLA region and in two other intergenic regions. Although these results need further confirmation in larger and independent samples, our findings in other SADs indicate that these genetic variants could potentially impact MCTD pathology by changing DNAm levels, as it has been previously suggested for other diseases and phenotypes (19, 21, 29). Future studies including larger samples and applying causal inference statistical methods, such as Mendelian randomization, would further confirm the intermediary role of DNAm in MCTD genetic risk, as it has been demonstrated for other pathologies.

This is also the first time that DNAm levels at five different SADs is compared in the same study. Our results indicate, firstly, that a high degree of blood epigenetic sharing is found between MCTD, SjS, and SLE patients, but not between MCTD and SSc or RA as it could have been expected from the disease commonalities and diagnostic criteria. The interferon epigenetic signature observed for MCTD has been widely reported in the EWAS literature for SLE and SjS in whole blood, but also in other different fractionated blood cell types (14–19, 24). Intriguingly, we show here that for MCTD a more profound epigenetic differentiation is observed between cases and controls, which could imply a molecular mechanisms underlying a more profound molecular changes. If these changes relate to clinical manifestations or symptoms would require further investigation.

Finally, we illustrate for the first time the great diagnostic potential of whole blood DNAm levels to discriminate MCTD patients. The results of our study show that DNAm levels at many individual MCTD-DMS in whole blood present very high diagnostic value to differentiate MCTD patients from healthy controls and perform reasonably well to discriminate MCTD from RA and SSc patients. DNAm levels at *PARP9* and *IFI44L* genes exhibit the highest discrimination ability, which is in line with findings from previous studies for other SADs and in other blood fractionated cell types (25, 30). Moreover, we show that adding the information of the top 10 discriminating CpG-sites boosts the diagnostic value and makes epigenetic biomarkers a potential tool to discriminate MCTD from SLE and SjS patients as well, even if they show a similar epigenetic signature. Given our promising results, and the fact that whole blood DNAm changes are easily measured from readily accessible peripheral blood samples, we conclude that epigenetic based biomarkers are ideal for novel biomarker development to diagnose and evaluate MCTD.

## Materials and Methods

### Subjects and Samples

Samples included in this study were obtained from the European PRECISESADS project [URL: http://www.precisesads.eu/] (31), a multi-center cross-sectional clinical study, with recruitment performed between December 2014 and October 2017 at 19 institutions in 9 countries (Austria, Belgium, France, Germany, Hungary, Italy, Portugal, Spain, and Switzerland, see Supplementary Note for a relation of centers involved in recruitment). Patients included in this study were aged 18 years or older, and diagnosed as having one of the following SADs: RA, SSc, SjS, SLE, and/or MCTD. Each patient was diagnosed according to the prevailing international classification criteria established for each of these diseases (6, 32–34). Patients that meet diagnostic criteria for more than one SADs were excluded from PRECISESADs project. Healthy individuals, i.e., not having any history of autoimmune or infectious diseases, were included as controls, and matched to cases to the extent possible, in gender, age and clinical center of origin. At the time of blood sampling, clinical and demographic information was obtained for every subject including treatment assessment.

A consensual protocol and informed consent was approved for by local ethics committees of each participating clinical center. All subjects provided written informed consent according to the declaration of Helsinki. For this work, we included patients for whom there was available DNA methylation data and/or genotypic information (see Supplementary Table 1).

### Genotyping and Imputation

Genomic DNA from whole blood was obtained by standard methods. All samples were genotyped using HumanCore−12-v1-0-B, InfiniumCoreExome-24v1-2, and InfiniumCoreExome-24v1-3 (Illumina, San Diego, CA, USA). Quality control (QC) was performed using PLINK v1.90b3.39 (35). A total of 213,234 variants passed our filtering criteria when including all subjects from PRECISESADs projects. We used the FRAPPE software that use single-nucleotide polymorphisms to estimate the global ancestry of each individual. To do this, an independent set of 2,706 genetic markers which exhibit substantially different frequencies between different populations, i.e., ancestry informative markers (AMs), were used. Also, all 2,504 individuals from the 1,000 Genomes Project (1000G) were included in the estimation of the ancestry as reference panel of the five main global populations (Africa, Europe, American, East Asian, and South Asian).

Genetic markers were removed following this criteria: (i) call rate < 90%, (ii) significant differential missingness between cases and controls (*P*-value < 1 × 10^−4^), and/or (iii) significant deviation from Hardy-Weinberg equilibrium (*P*-value < 0.01 in controls and *P*-value < 1 × 10^−4^ in cases). Samples were excluded from the study following this criteria: (i) call rate < 98% and also a high heterozygosity rate, i.e., 6 standard deviations from the centroid; (ii) duplicated or related individuals were identified using identity-by-descent criteria with REAP (36), (iii) a kinship coefficient < 0.25, (iv) clinical gender data did not match genotypic data, and finally, (v) <55% European ancestry.

Inference methods based on linkage disequilibrium structure were used in order to increase the number of genetic markers. Imputation was performed using the Michigan Imputation Server [URL: https://imputationserver.sph.umich.edu/index.html] (37) and Haplotype Reference Consortium (HRC) as reference panel [URL: http://www.haplotype-reference-consortium.org/] (38) and using a rsq cutoff to filter the imputed genotypes of 0.7. Imputed variants were also filtered according to the protocol described above.

### Genome-Wide DNA Methylation Profiling

After DNA extraction from whole peripheral blood and bisulfite conversion, the genome for each sample was amplified, fragmented and hybridized to the corresponding Illumina arrays according to the manufacturer's protocol. The QC of samples, probes, and the normalization of the data were performed using the meffil R software following the developers' guidelines (39). Samples were excluded based on the detection *p*-value criteria > 99%, poor bisulfite conversion based on control dashboard check, sex mismatches according to failed chromosome X and Y clustering, and failure to match genotypic information. Probes were filtered out based on detection *p*-value > 0.01 in > 95% of samples. Additionally, all probes located at the X and Y chromosomes were separated to avoid gender bias. Probes with genetic variants at their CpG sites with a minor allele frequencies higher than 0.05 and those that map to multiple genomic regions were also excluded following the instructions of recent works that evaluate the Illumina Methylation EPIC BeadChip microarray (40). After QC steps, we obtained information on 776,398 probes for samples profiled using the Infinium Methylation EPIC BeadChip (Illumina, San Diego, CA, USA). Additionally, we obtained DNAm data that were profiled using the Infinium Methylation 450K BeadChip (Illumina, San Diego, CA, USA), for which we obtained trustable information on 433,337 probes.

The raw methylation beta values were background corrected and normalized using the functional normalization. DNAm was measured as a beta value ranging from 0 to 1. Zero represents an unmethylated state (0% molecules methylated at a particular sites) while 1 represents a fully methylated state (100% molecules methylated). We also estimated the cellular composition of whole blood from the DNAm profiles using the Houseman method and the blood gse35069 as a cell reference panel (41). Estimated proportions for neutrophils, monocytes, B lymphocytes, CD4+ lymphocytes, CD8+ lymphocytes, and natural killer (NK) cells were used as covariates for subsequent association analyses. For these procedures we used again the meffil R-package (39).

### Identification of MCTD-Associated Genome-Wide DNA Methylation Patterns

In order to maximize our findings, we separated our samples accordingly to the platform used to profile DNAm. As a discovery sample we included all subjects with DNAm data based on the Infinium Methylation EPIC BeadChip, which comprised 31 subjects diagnosed with MCTD and 255 healthy controls (CTRL). As a replication sample, we included 21 MCTD patients and 124 healthy controls for which we had DNAm data based on the Infinium Methylation 450K BeadChip. Most of the probes included in the 450K array (~90%) are also included in the EPIC array. Epidemiological and clinical features of the samples included in the discovery and the replication samples are summarized in Supplementary Table 1. Firstly, we performed epigenome-wide association analysis (EWAS) to identify MCTD-associated differential methylated positions (MCTD-DMS) along the genome. For every CpG in our discovery sample we evaluated the effect of being diagnosed with MCTD on DNAm using a linear regression model corrected by age, sex, batch effects and estimated cell proportions. We also searched for CpG-sites showing DNAm variance differences between MCTD patients and healthy samples and called these as MCTD-associated variable methylated CpG-sites (MCTD-VMS). For that, we first residualized DNAm levels correcting for all covariates in a linear regression model. Then, we searched for variance differences in DNAm residuals between cases and controls by applying a Levene's test that accounts for mean differences. A Bonferroni correction was used to declare genome-wide significance (*P* < 6.25 × 10^−8^) and to identify MCTD-DMS and MCTD-VMS. For each MCTD-DMS and MCTD-VMS the mean and the variance was calculated in MCTD patients and CTRL. The same analyses and models were used in the replication sample to evaluate the robustness of significant associations in an independent cohort. We established as replicated MCTD-DMS and MCTD-VMS as those showing consistent effect direction and P < 0.05.

In order to investigate the influence of treatment effects in our results, we also run EWAS adjusted the linear regression model by variables representing whether or not individuals were under specific treatments at the time of blood sampling. We only included those treatments that were prescribed in more than three patients, which are steroids (*N* = 12), antimalarial (*N* = 13), and/or immunosuppressive drugs (*N* = 9) (see Supplementary Table 1). Furthermore, we perform stratified analyses by treatment with the aim to identify treatment-specific effects at MCTD-DMS. For that we run the linear models separately in treated and untreated samples and look for those CpG sites that are significant after Bonferroni correction in one group (*P* < 2.7 × 10^−04^) but with little evidence of association in the other (*P* > 0.01). In order to give robustness to treatment specific effects, we performed the same analyses in our replication sample. All statistical analyses were performed using R (v3.4.2) [URL: http://www.R-project.org/].

### Functional Enrichment

Significant MCTD-DMS were annotated to genes and gene locations according to annotation files provided by Illumina, from which we obtained a list of unique differentially methylated genes. The online tool of Enrichr program was used to perform gene set enrichment analysis based on gene ontology (GO) terms (42).

### Methylation Quantitative Trait Loci (meQTL) Analysis

In order to find *cis*-genetic variation affecting DNAm levels at MCTD-DMS, meQTL analysis was performed. Linear regression models were applied to evaluate the effect of all SNPs sitting closer than 1 Mb to the evaluated CpG site, on DNAm levels using the Matrix eQTL R software (43). The models were corrected for sex, age, batch effects, cellular proportions, disease status and the first genetic principal component. Only common SNPs with a MAF > 0.05 were included in the analyses. We discovered meQTLs in a sample that comprised 259 subjects for which we had both EPIC DNAm data and genotypic information. We used a FDR < 0.05 to correct for multiple testing. We replicated our findings in a sample that comprised 116 subjects for which we had both 450 K data and genotypic information.

### Genetic Associations

Genetic association case-control analyses were conducted using PLINK v1.9 (35). Logistic regressions under the additive model were conducted to interrogate the association between genetic variants involved in MCTD-meQTLs and SAD diagnosis. For that, we compared the allele frequencies of 428 SLE, 400 SSc, 395 SjS, 380 RA, 103 PAPS, and 89 MCTD patients samples vs. 570 controls. We show none or little evidence for genetic stratification as detected by genomic control coefficient (GC) (1.20 < GC < 1). We used a *P*-value < 0.05 to report suggestive genetic associations.

### Cross-SADs Comparative Epigenetic Analyses

We used linear regression models to find differentially methylated CpG-sites associated with other SADs for which we had information on DNAm EPIC data. We included 234 SLE, 206 SjS, 217 RA, and 177 SSc patients and the same linear models than previously explained (Supplementary Table 1). The effect sizes obtained for each disease were compared with that obtained for MCTD by means of Pearson's correlation. We searched for significant DNAm differences between MCTD and the other SADs by performing cases vs. cases analyses in a linear regression model framework that included the same covariates as previously explained.

### Binary Prediction Analysis and ROC Evaluation

The diagnostic utility of all MCTD-DMS was evaluated by performing logistic regression analyses and calculating the AUC curve in both our discovery and replication sample using the pROC R package. We performed logistic regression models to find out how well epigenetic markers differentiate MCTD from the healthy population and also from every other SADs included in this study. The covariates used in the logistic models were the same as previously explained for linear models.

## Data Availability

All datasets generated for this study are included in the manuscript and the Supplementary Files.

## Author Contributions

EC-M, MA-R, and MT contributed to the conception and design of the study and also drafted the manuscript. The PRECISESADS Clinical Consortium members agreed on the clinical data, recruited the patients, and performed their detailed clinical assessment. EC-M, GB, EP, MK, and MM-B contributed to analyzing the data. MA-R, JM, and EB contributed to data generation and sample recruitment. All authors read and approved the final manuscript.

### Conflict of Interest Statement

The authors declare that the research was conducted in the absence of any commercial or financial relationships that could be construed as a potential conflict of interest.
