# Supplementary material for: Epigenome-Wide Comparative Study Reveals Key Differences Between Mixed Connective Tissue Disease and Related Systemic Autoimmune Diseases
Source: Front Immunol. 2019 Aug 7;10:1880. doi: 10.3389/fimmu.2019.01880 (PMC6693476; doi:10.3389/fimmu.2019.01880)

Supplementary Figure 1 | Quantile-quantile plot for MCTD epigenome-wide association study results

The observed log10 P value obtained in the EWAS linear regression model for each CpG site analysed is depicted in the *y*-axis against the null distribution of expected log10 P values in the *x*-axis

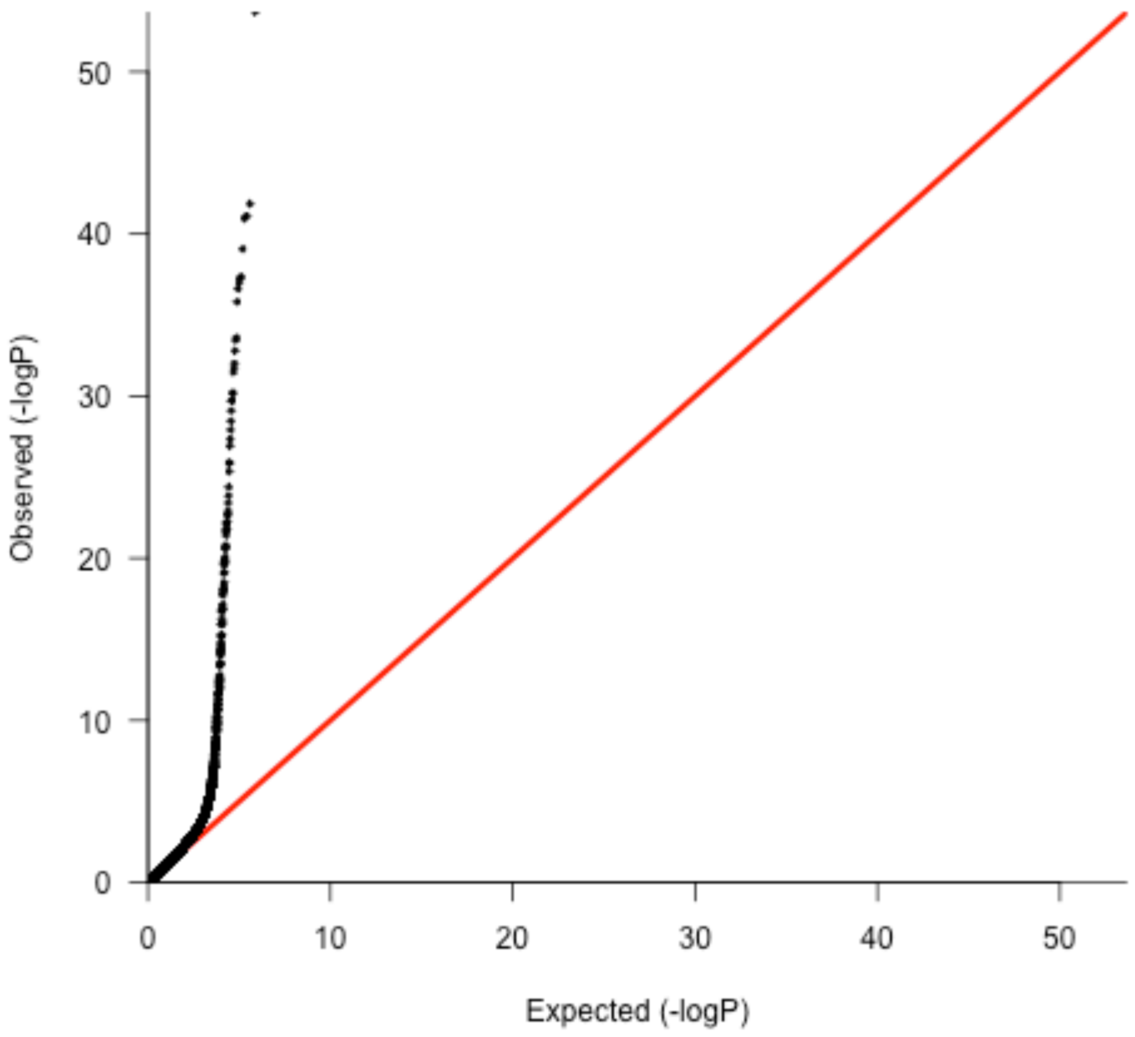

Supplementary Figure 2 | Quantile-quantile plot for meQTL results

The observed log10 P value obtained in the linear regression model for each SNP-CpG association in meQTL analysis is depicted in the y-axis against the null distribution of expected log10 P values in the *x*-axis

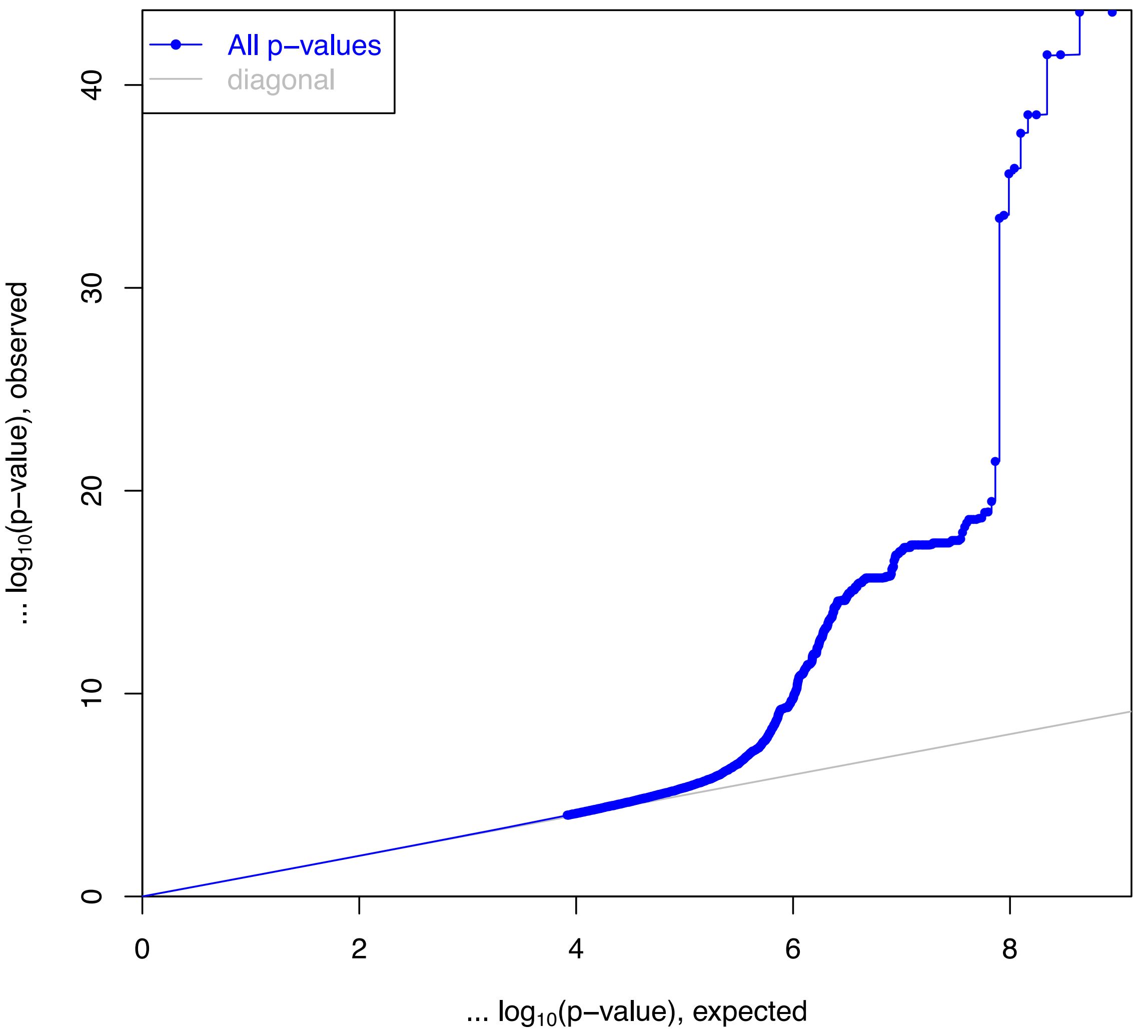

Supplementary Figure 3 | Comparison of results for MCTD-associated epigenetic signals in other SADs in the replication cohort

Correlation plots comparing the effect effect sized obtained in the MCTD epigenetic association results (in the *y*-axis) with those obtained for other systemic autoimmune diseases (*x*-axis) in the 450K methylation platform

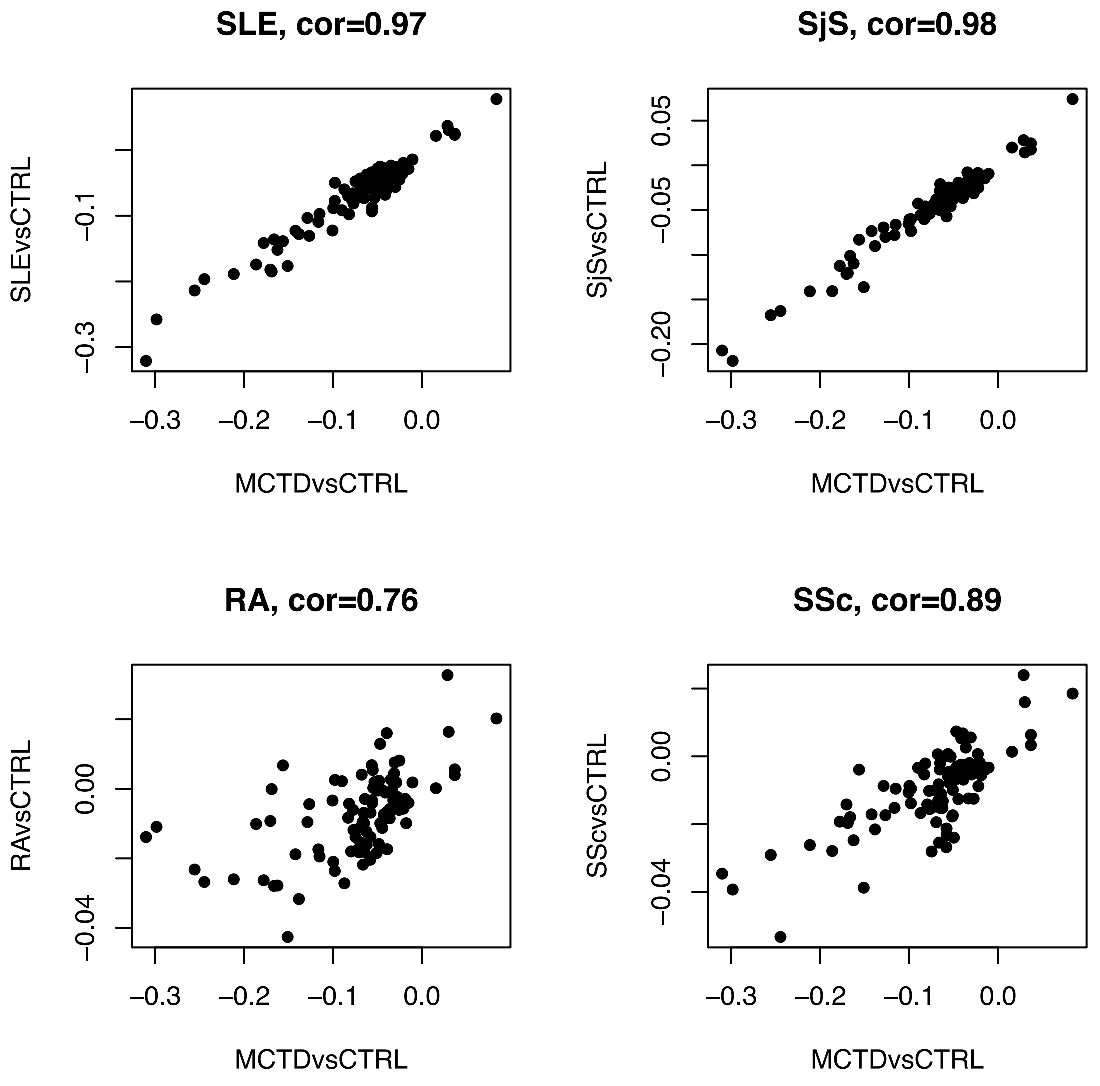

Supplement: Supplementary file 2 [file Data_Sheet_1.PDF]
