# Supplementary material for: Epigenome-Wide Comparative Study Reveals Key Differences Between Mixed Connective Tissue Disease and Related Systemic Autoimmune Diseases
Source: Front Immunol. 2019 Aug 7;10:1880. doi: 10.3389/fimmu.2019.01880 (PMC6693476; doi:10.3389/fimmu.2019.01880)
Supplement: Supplementary file 3 [file Table_3.DOCX]

Lorenzo Beretta1, Barbara Vigone^[[1]](#endnote-1)^,

Jacques-Olivier Pers2, Alain Saraux2, Valérie Devauchelle-Pensec2, Divi Cornec2, Sandrine Jousse-Joulin ^[[2]](#endnote-2)^,

Bernard Lauwerys^3^, Julie Ducreux^3^, Anne-Lise Maudoux^[[3]](#endnote-3)^,

Carlos Vasconcelos^4^, Ana Tavares^4^, Esmeralda Neves^4^, Raquel Faria ^[[4]](#endnote-4)^, Mariana Brandão^4^,

Ana Campar^4^, António Marinho^4^, Fátima Farinha^4^, Isabel Almeida^4^

Miguel Angel Gonzalez-Gay Mantecón5, Ricardo Blanco Alonso5, Alfonso Corrales Martínez^[[5]](#endnote-5)^,

Ricard Cervera^6^, Ignasi Rodríguez-Pintó^6^, Gerard Espinosa ^[[6]](#endnote-6)^,

Rik Lories7, Ellen De Langhe^[[7]](#endnote-7)^,

Nicolas Hunzelmann8, Doreen Belz^[[8]](#endnote-8)^,

Torsten Witte9, Niklas Baerlecken ^[[9]](#endnote-9)^,

Georg Stummvoll^10^, Michael Zauner^10^, Michaela Lehner ^[[10]](#endnote-10)^,

Eduardo Collantes^11^, Rafaela Ortega-Castro^11^, Mª Angeles Aguirre-Zamorano^11^, Alejandro Escudero-Contreras^11^, Mª Carmen Castro-Villegas ^[[11]](#endnote-11)^

Norberto Ortego^12^, María Concepción Fernández Roldán ^[[12]](#endnote-12)^,

Enrique Raya^13^, Inmaculada Jiménez Moleón ^[[13]](#endnote-13)^,

Enrique de Ramon^14^, Isabel Díaz Quintero^[[14]](#endnote-14)^,

Pier Luigi Meroni^15^, Maria Gerosa^15^, Tommaso Schioppo^15^, Carolina Artusi^[[15]](#endnote-15)^,

Carlo Chizzolini^16^, Aleksandra Zuber^16^, Donatienne Wynar^[[16]](#endnote-16)^,

Laszló Kovács^17^, Attila Balog^17^, Magdolna Deák^17^, Márta Bocskai^17^, Sonja Dulic^17^, Gabriella Kádár^[[17]](#endnote-17)^,

Falk Hiepe^18^, Velia Gerl^18^, Silvia Thiel^[[18]](#endnote-18)^,

Manuel Rodriguez Maresca^19^, Antonio López-Berrio^19^, Rocío Aguilar-Quesada^19^, Héctor Navarro-Linares ^[[19]](#endnote-19)^

**Affiliations**

1. Referral Center for Systemic Autoimmune Diseases, Fondazione IRCCS Ca’ Granda Ospedale Maggiore Policlinico di Milano, Italy. [↑](#endnote-ref-1)
2. Centre Hospitalier Universitaire de Brest, Hospital de la Cavale Blanche, Brest, France. [↑](#endnote-ref-2)
3. Pôle de pathologies rhumatismales systémiques et inflammatoires, Institut de Recherche Expérimentale et Clinique, Université catholique de Louvain, Brussels, Belgium. [↑](#endnote-ref-3)
4. Centro Hospitalar do Porto, Portugal. [↑](#endnote-ref-4)
5. Servicio Cantabro de Salud, Hospital Universitario Marqués de Valdecilla, Santander, Spain. [↑](#endnote-ref-5)
6. Hospital Clinic I Provicia, Institut d’Investigacions Biomèdiques August Pi i Sunyer, Barcelona, Spain. [↑](#endnote-ref-6)
7. Katholieke Universiteit Leuven, Belgium. [↑](#endnote-ref-7)
8. Klinikum der Universitaet zu Koeln, Cologne, Germany. [↑](#endnote-ref-8)
9. Medizinische Hochschule Hannover, Germany. [↑](#endnote-ref-9)
10. Medical University Vienna, Vienna, Austria. [↑](#endnote-ref-10)
11. Servicio Andaluz de Salud, Hospital Universitario Reina Sofía Córdoba, Spain. [↑](#endnote-ref-11)
12. Servicio Andaluz de Salud, Complejo hospitalario Universitario de Granada (Hospital Universitario San Cecilio), Spain. [↑](#endnote-ref-12)
13. Servicio Andaluz de Salud, Complejo hospitalario Universitario de Granada (Hospital Virgen de las Nieves), Spain. [↑](#endnote-ref-13)
14. Servicio Andaluz de Salud, Hospital Regional Universitario de Málaga, Spain [↑](#endnote-ref-14)
15. Università degli studi di Milano, Milan, Italy. [↑](#endnote-ref-15)
16. Hospitaux Universitaires de Genève, Switzerland. [↑](#endnote-ref-16)
17. University of Szeged, Szeged, Hungary. [↑](#endnote-ref-17)
18. Charite, Berlin, Germany. [↑](#endnote-ref-18)
19. Andalusian Public Health System Biobank, Granada, Spain [↑](#endnote-ref-19)
